# Early transcriptional events linked to induction of diapause revealed by RNAseq in larvae of drosophilid fly, *Chymomyza costata*

**DOI:** 10.1186/s12864-015-1907-4

**Published:** 2015-09-21

**Authors:** Rodolphe Poupardin, Konrad Schöttner, Jaroslava Korbelová, Jan Provazník, David Doležel, Dinko Pavlinic, Vladimír Beneš, Vladimír Koštál

**Affiliations:** Biology Centre CAS, Institute of Entomology, Branišovská 31, 37005 České Budějovice, Czech Republic; Faculty of Science, University of South Bohemia, Branišovská 31, 37005 České Budějovice, Czech Republic; Genomics Core Facility, European Molecular Biology Laboratory, Meyerhofstraße 1, 69117 Heidelberg, Germany

**Keywords:** Fruit fly, Photoperiodism, Development, Dormancy, Gene expression, Cell cycle, Ecdysteroids, Epigenetics factors

## Abstract

**Background:**

Diapause is a developmental alternative to direct ontogeny in many invertebrates. Its primary adaptive meaning is to secure survival over unfavourable seasons in a state of developmental arrest usually accompanied by metabolic suppression and enhanced tolerance to environmental stressors. During photoperiodically triggered diapause of insects, the ontogeny is centrally turned off under hormonal control, the molecular details of this transition being poorly understood. Using RNAseq technology, we characterized transcription profiles associated with photoperiodic diapause induction in the larvae of the drosophilid fly *Chymomyza costata* with the goal of identifying candidate genes and processes linked to upstream regulatory events that eventually lead to a complex phenotypic change.

**Results:**

Short day photoperiod triggering diapause was associated to inhibition of 20-hydroxy ecdysone (20-HE) signalling during the photoperiod-sensitive stage of *C. costata* larval development. The mRNA levels of several key genes involved in 20-HE biosynthesis, perception, and signalling were significantly downregulated under short days. Hormonal change was translated into downregulation of a series of other transcripts with broad influence on gene expression, protein translation, alternative histone marking by methylation and alternative splicing. These changes probably resulted in blockade of direct development and deep restructuring of metabolic pathways indicated by differential expression of genes involved in cell cycle regulation, metabolism, detoxification, redox balance, protection against oxidative stress, cuticle formation and synthesis of larval storage proteins. This highly complex alteration of gene transcription was expressed already during first extended night, within the first four hours after the change of the photoperiodic signal from long days to short days. We validated our RNAseq differential gene expression results in an independent qRT-PCR experiment involving wild-type (photoperiodic) and NPD-mutant (non-photoperiodic) strains of *C. costata*.

**Conclusions:**

Our study revealed several strong candidate genes for follow-up functional studies. Candidate genes code for upstream regulators of a complex change of gene expression, which leads to phenotypic switch from direct ontogeny to larval diapause.

**Electronic supplementary material:**

The online version of this article (doi:10.1186/s12864-015-1907-4) contains supplementary material, which is available to authorized users.

## Background

Diapause is an environmentally programmed and hormonally regulated period of dormancy in invertebrates [1, 2]. Why study diapause? In an attempt to answer this question, Denlinger [3] provides various good explanations. First and foremost, diapause represents a central part of the life-cycle in many species and secures their survival over unfavourable seasons [3, 4]. More generally, diapause offers an excellent possibility to probe fundamental questions about the regulation of organismal development [3]. Environmental seasonal cues, most often photoperiod, are perceived during a species-specific sensitive stage and transduced into one of two ontogenetic alternatives: direct development versus diapause development [5]. During diapause development, the active lifestyle and the ontogenetic processes are transiently (for the harsh season) but dramatically turned off. The cell division cycles in proliferative tissues stop, the growth and morphogenesis slow down or cease, and, in adults, the development of gametes and reproduction behaviour disappear [6–8]. In contrast, other processes might be bolstered, such as metabolic pathways linked to the building of energy reserves [9, 10], and cellular protective systems to prevent aging [11], oxidative damage [12], or injury caused by a wide range of environmental stressors linked to harsh seasons [4, 13, 14]. The period of developmental arrest during diapause is not static but rather represents a dynamic succession of more or less clearly expressed ecophysiological phases of induction, preparation, initiation, maintenance and termination [6].

Despite the indisputable importance of diapause for insect science/applications and its great potential for basic research, molecular and physiological regulation of diapause remains mostly unknown [15]. This is partially caused by the fact that diapause is very weakly expressed in the classic model insect, *Drosophila melanogaster* [16]. Nevertheless, it is well established that switching between direct development and diapause is controlled by the alteration of hormonal signalling. In most insects, the decrease or absence of secretion of basic developmental hormones, juvenoids and ecdysteroids induces diapause [17–19]. Very briefly, these hormones are synthesized in endocrine glands, *corpora allata* and prothoracic gland, respectively, which are under the control of neuropeptides produced by specific brain neurons [20, 21], which, in turn, receive information about environmental cues from sensory neurons or light-sensitive cells including, most probably, central circadian clock neurons [22–24]. Recent discoveries in mosquito, *Culex pipiens* and fruit fly, *D. melanogaster* indicate that this transduction pathway communicates with insuling signalling pathway [25–27] but the details of this crosstalk only start to emerge and will need verification in other species.

Increasing accessibility of omic technologies and their applicability to non-model insects brought an important momentum for diapause research [28]. Many authors used transcriptomics to characterise gene expression changes in response to diapause. Their major motivations were twofold: to describe a holistic picture of the complex diapause syndrome and to reveal specific candidate genes and processes that critically regulate diapause transitions. These aims are complicated by the fact that different insect species enter diapause in different ontogenetic stages (embryos, larvae, pupae and adults), which widely differ in their hormonal milieu and tissue complexity. Although the common phenotypic features of diapause (developmental arrest, metabolic suppression, environmental stress resistance, etc.) are similar in most species, there may be diverse transcriptional strategies for producing them [14]. Most previous studies on transcriptomic signatures of insect diapause compared the nondiapausing insects to diapausing individuals in which the diapause syndrome was already fully expressed in the phase of diapause maintenance [29–39]. In other studies, the insects were exposed to stimuli known to terminate diapause and their transition from diapause to post-diapause quiescence and/or the resumption of development was studied [40–43]. Some previous studies specifically addressed the events during the initiation phase of diapause, alternatively called “early” diapause [14, 42, 44–49], and only very few studies focused on the earliest phase of diapause induction. The study by [50], for instance, detected only a few transcripts that were differentially regulated (4 upregulated, 22 downregulated) in the heads of photoperiod-sensitive generation of pea aphids, *Acyrthosiphon pisum* in response to acute shortening of daylength, which induces the egg diapause in the offspring of after-next generation. In contrast, the recent study [51] of maternal diapause induction in the mosquito *Aedes albopictus* identified differential expression of 2251 genes in response to diapause-inducing short-day photoperiods. Some other papers examined induction-linked expression in a limited number of genes only. For instance, we [7] previously assessed changes in the relative abundances of seven genes coding for cell cycle regulatory factors in the photoperiod-sensitive 3rd instar larvae of *Chymomyza costata*, and [52] studied eight genes related to small RNA biogenesis in the photoperiod-sensitive 1st instar larvae of the flesh fly, *Sarcophaga bull*ata. However, the most important regulatory events during early stages of diapause induction, which decide about the future developmental destiny, remained underexplored.

In this study, we characterize global patterns of gene expression associated with very early stages of diapause in the larvae of drosophilid fly, *Chymomyza costata* using RNAseq technology. Our main goal was to identify candidate genes and processes linked to the regulation of the diapause *induction* phase (*sensu* [6]), when the decision for diapause development has just been taken but the diapause phenotype is not yet expressed. We previously found that the first three days of the 3rd larval instar is a period of maximum sensitivity to daylength in *C. costata*. Irrespective of photoperiodic conditions (long-day, LD or short-day, SD), early 3rd instar larvae actively move, feed, grow and develop while at the same time they sense the photoperiod and determine their future developmental destiny accordingly (direct development under LD vs. larval diapause under SD). The typical diapause traits will occur in *C. costata* larvae later, during the phase of diapause *initiation* (at age of 10–40 days of 3rd instar) [53, 54]. In addition to comparing larvae reared under constant LD and SD photoperiods from early embryonic stage, we also transferred the larvae from LD to SD conditions during their sensitive stage and observed immediate transcriptomic responses (within one hour and four hours) that might be linked to the *developmental switch* from direct development to diapause. Although much of the work we present is descriptive, our comparison of gene expressions in the photoperiodic wild-type (Sapporo) strain and non-photoperiodic diapause (NPD) mutant strain, which lacks the ability to enter photoperiod-induced diapause, allows us to deduce the potential changes or disturbances linked to light OFF (present in both strains) from the directed developmental response to daylength (present in wild-type strain only).

## Results

### De-novo *C. costata* transcriptome assembly

In our paired end sequencing, we obtained 475,801,314 reads in total and 9 % of reads were removed after quality filtering. Our SOAP *de novo* assembly returned a total of 113,446 *C. costata* sequences. The N25, N50 and N75 values were 3900 nt, 1706 nt and 463 nt, respectively. The average GC content was 39.40 %. After running Blast2GO, 23,315 sequences showed blastX hits and 18,718 sequences were assigned with at least one GO-term. Following DESeq2 analysis, only transcripts with the baseMean > 10 were considered, which reduced the number of sequences to 21,327 putative mRNA transcripts (see Additional file 1: Table S1 for the list of putative transcripts and the results of the analysis).

### Differentially expressed transcripts in relation to developmental destiny (SD vs. LD)

In our single end sequencing experiment, we obtained 26.6 ± 5.9 mil (mean ± SD) reads per library. A very light filtering was applied by removing in average 0.20 % of contaminant sequences, mainly adapters. The percentages of reads mapped to our reference transcriptome were all above 97 %.

**Fig. 1:**
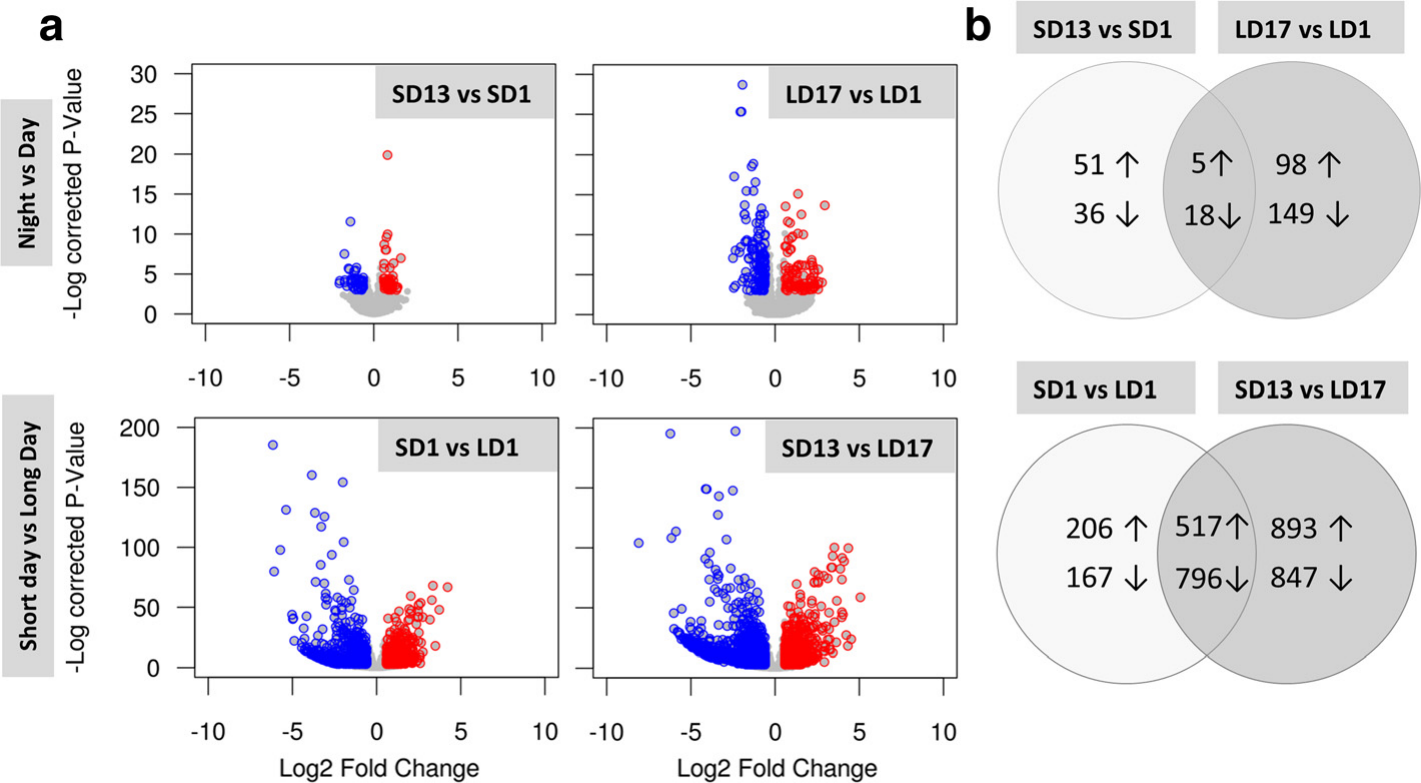
Differential gene expression in larvae of *Chymomyza costata* during Night vs. Day and under Short Day vs. Long Day (SD) photoperiodic conditions. The larvae were sampled at two different Zeitgeber times: Zt1, one hour after lights ON; Zt13 (Zt17), one hour after lights OFF at SD (LD) conditions (see Fig. 5 for schematic depiction). The sequences showing statistically significant up- and down-regulations (log2 fold change above 0.55 or below −0.55 and a corrected *P*-value below 0.01) are highlighted in (**a**) volcano plots (up, red; down, blue) and (**b**) Venn’s diagrams (up, ↑; down, ↓)

First, we compared gene expression patterns in young 3rd instar wild-type larvae that were continuously reared under LD or SD regimes. The potential effect of diurnal oscillations on gene expression was partially controlled by sampling the larvae at two different Zeitgeber times (Zt) corresponding to one hour (1 h) after light ON (Zt1) and 1 h after light OFF (Zt17 at LD, Zt13 at SD). The shapes of volcano plots (Fig. 1) suggest that there was much more variation between SD vs. LD compared to Night vs. Day. For the Night vs. Day comparison, 270 transcripts were found differentially expressed (FC > 1.5; *P* < 0.05) under LD conditions and 110 transcripts were differentially expressed under SD conditions. Only 23 Night vs. Day differentially expressed transcripts were shared between LD and SD photoperiodic regimes (Fig. 1a). Most of these 23 transcripts (Additional file 2: Table S2) were downregulated during night with a high proportion of transcripts linked with cuticle.

For the SD vs. LD comparison, 1686 transcripts were found differentially expressed during day (Zt1) while 3053 transcripts were differentially expressed during night (Zt13 or Zt17, respectively), and 1313 differentially expressed transcripts were shared between day and night conditions (Fig. 1b). The enrichment analysis identified several clusters of GO terms to be over-represented in the genes that were up- or downregulated in SD vs. LD (Table 1). The SD-upregulated processes involved metabolism of lipids, amino acids, organic acids and development of chitin-based cuticle, while the SD-downregulated processes were linked to development, cell division cycle and DNA replication.

**Table 1:** Clusters of GO terms and KEGG pathways found significantly over represented in *Chymomyza costata* larvae exposed to Short day vs. Long day photoperiodic conditions

Short day vs. Long day (SD vs. LD)		
GOID	GOTerm	Cor. *P*-value
Up regulated genes		
Metabolism of lipids		
GO:0004806	Triglyceride lipase activity	1,30E-05
GO:0006629	Lipid metabolic process	7,86E-03
GO:0016298	Lipase activity	2,10E-08
GO:0016788	Hydrolase activity, acting on ester bonds	4,26E-04
GO:0052689	Carboxylic ester hydrolase activity	3,72E-04
Metabolism of amino acids		
GO:0008483	Transaminase activity	2,60E-02
GO:0016769	Transferase activity, transferring nitrogenous groups	2,60E-02
Metabolism of organic acids and detoxification		
GO:0019752	Carboxylic acid metabolic process	2,68E-03
GO:0006520	Cellular amino acid metabolic process	3,65E-03
KEGG:00980	Metabolism of xenobiotics by cytochrome P450	3,92E-03
GO:0006082	Organic acid metabolic process	4,23E-03
GO:0043436	Oxoacid metabolic process	4,23E-03
Activity of glycosidases, glycosylation		
GO:0004553	Hydrolase activity, hydrolyzing O-glycosyl compounds	9,36E-03
GO:0004559	Alpha-mannosidase activity	2,60E-02
GO:0015923	Mannosidase activity	3,04E-02
GO:0016798	Hydrolase activity, acting on glycosyl bonds	2,72E-03
Cuticle development		
GO:0005214	Structural constituent of chitin-based cuticle	5,53E-03
GO:0008010	Structural constituent of chitin-based larval cuticle	4,23E-03
GO:0040003	Chitin-based cuticle development	2,66E-02
Down regulated genes		
Organismal development		
GO:0002165	Instar larval or pupal development	1,39E-02
GO:0009791	Post-embryonic development	9,46E-03
GO:0007275	Multicellular organismal development	1,19E-04
GO:0030154	Cell differentiation	3,38E-03
GO:0048869	Cellular developmental process	8,98E-03
Cell cycle		
GO:0007049	Cell cycle	1,20E-07
GO:0000278	Mitotic cell cycle	4,60E-07
GO:0022402	Cell cycle process	4,66E-07
GO:0000280	Nuclear division	9,14E-07
GO:0048285	Organelle fission	1,35E-06
DNA replication		
KEGG:03030	DNA replication	1,68E-04
GO:0006259	DNA metabolic process	3,87E-02
GO:0006260	DNA replication	1,87E-03
GO:0042023	DNA endoreduplication	1,79E-02
GO:0044786	Cell cycle DNA replication	6,75E-03
Cation symporter activity		
GO:0015293	Symporter activity	2,63E-02
GO:0015294	Solute:cation symporter activity	2,11E-02
Activity of peptidases		
KEGG:00480	Glutathione metabolism	1,35E-02
GO:0004177	Aminopeptidase activity	2,80E-03

### Differentially expressed transcripts in relation to developmental switch (T vs. LD)

**Fig. 2:**
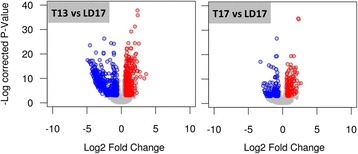
Differential gene expression in larvae of *Chymomyza costata* that were transferred (T) from Long Day (LD) to Short Day (SD) photoperiodic conditions. The larvae were sampled at two Zeitgeber times, Zt13 and Zt17 (see Fig. 5 for schematic depiction). Volcano plots highlight the sequences showing statistically significant up-(red) and down-(blue) regulations (log2 fold change above 0.55 or below −0.55 and a corrected *P*-value below 0.01)

Next, we compared the gene expression patterns in young 3rd instar wild-type larvae in which the switch of developmental destiny from direct development to diapause was initiated by transferring (T) them from LD to SD conditions. Large variation in gene expression was observed between T13 vs. LD17 (421 up, 482 down), while the comparison of T17 vs. LD17 showed smaller variation (130 up, 150 down) (Fig. 2). The GO term enrichment analysis (Table 2) revealed that the transfer from LD to SD conditions was linked with upregulation of genes involved in nascent protein folding and processing in endoplasmic reticulum, and with the stress response mediated by heat-shock proteins. In addition, the transfer to SD conditions resulted in upregulation of processes linked to metabolism of lipids, organic acids, and cuticle development. In contrast, downregulation was observed in several processes that indicate the blockade of active development: microtubular transport, spermatogenesis, mRNA processing, and the enzymatic complex of mitochondrial TCA cycle (Table 2).

**Table 2:** Clusters of GO terms and KEGG pathways found significantly over represented in *Chymomyza costata* larvae transferred from Long day to Short day photoperiodic conditions

Transfer Zt13 vs. Long day Zt 17 (T13 vs. LD17)		
GOID	GOTerm	Cor. *P*-value
Up regulated genes		
Protein processing in endoplasmic reticulum (ER)		
KEGG:04141	Protein processing in endoplasmic reticulum	2,54E-06
GO:0070972	Protein localization to endoplasmic reticulum	2,27E-04
GO:0006613	Cotranslational protein targeting to membrane	2,01E-03
GO:0006614	SRP-dependent cotranslational protein targeting to membrane	2,01E-03
GO:0045047	Protein targeting to ER	2,01E-03
Stress response and heat shock proteins		
GO:0006457	Protein folding	9,55E-03
GO:0009408	Response to heat	3,15E-02
Metabolism of lipids		
GO:0044255	Cellular lipid metabolic process	7,41E-04
Metabolism of organic acids and detoxification		
GO:0019752	Carboxylic acid metabolic process	1,40E-05
GO:0006082	Organic acid metabolic process	2,87E-05
GO:0043436	Oxoacid metabolic process	2,87E-05
GO:0032787	Monocarboxylic acid metabolic process	7,35E-04
GO:0006835	Dicarboxylic acid transport	3,16E-02
GO:0006839	Mitochondrial transport	2,75E-02
Cuticle development		
GO:0005214	Structural constituent of chitin-based cuticle	1,03E-12
GO:0008010	Structural constituent of chitin-based larval cuticle	3,34E-10
GO:0040003	Chitin-based cuticle development	9,86E-10
GO:0042335	Cuticle development	1,14E-09
Down regulated genes		
Microtubular transport		
GO:0007017	Microtubule-based process	1,77E-07
GO:0006928	Cellular component movement	9,25E-05
GO:0007018	Microtubule-based movement	6,98E-04
GO:0008017	Microtubule binding	9,64E-04
GO:0015631	Tubulin binding	1,75E-03
Development, Spermatogenesis		
GO:0007283	Spermatogenesis	1,31E-05
GO:0048232	Male gamete generation	1,38E-05
GO:0048515	Spermatid differentiation	5,59E-03
mRNA processing		
GO:0000340	RNA 7-methylguanosine cap binding	2,43E-02
GO:0016281	Eukaryotic translation initiation factor 4 F complex	4,37E-02
TCA metabolism		
GO:0030062	Mitochondrial tricarboxylic acid cycle enzyme complex	2,43E-02
GO:0045239	Tricarboxylic acid cycle enzyme complex	3,32E-02
Activity of peptidases		
GO:0004177	Aminopeptidase activity	7,02E-04
GO:0008238	Exopeptidase activity	7,27E-03

### Validation of RNAseq results

**Fig. 3:**
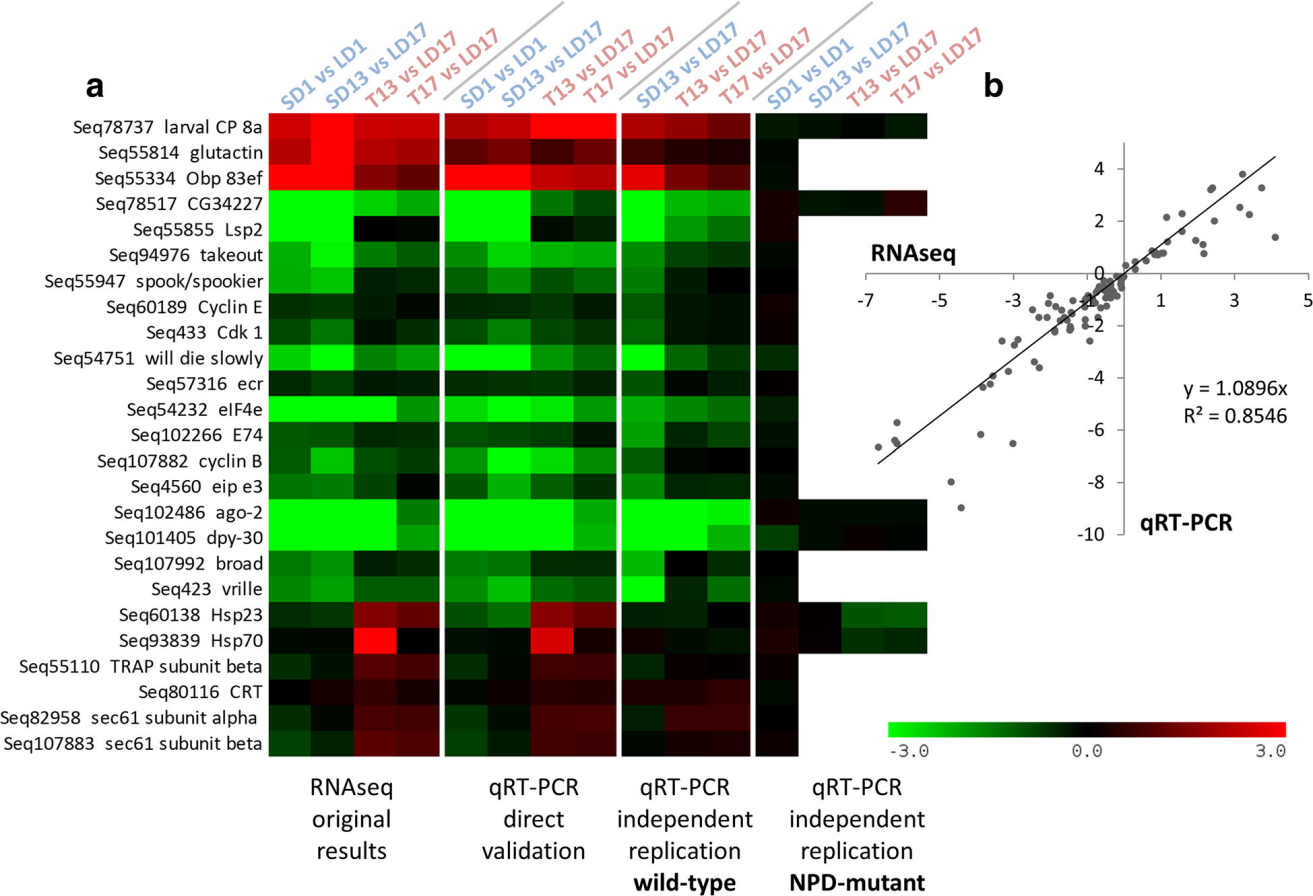
Results of three-step validation of RNAseq differential gene expression analysis. Each heat map (**a**) shows log2 fold transformed differences in mRNA relative abundances in 25 selected genes. The columns show (from left to right): original results obtained by DeSEQ2 analysis of RNAseq (Illumina) sequencing data; direct validation by qRT-PCR (using the same total RNA samples as used for RNAseq analysis); qRT-PCR analysis conducted on an independent replication of the experiment using wild-type and NPD-mutant *Chymomyza costata* larvae. The linear regression analysis (**b**) shows good correlation between RNAseq results and direct qRT-PCR validation. The larvae were exposed to Long Day (LD), Short Day (SD) and Transfer (T) from LD to SD photoperiodic conditions (see Fig. 5 for schematic depiction)

The left heat map in Fig. 3a shows selected results of the RNAseq analysis (data taken from Additional file 1: Table S1) while three other heat maps exhibit results of a three-step qRT-PCR validation assay in 25 selected transcripts. Direct validation (first step) showed a very good match between RNAseq and qRT-PCR methods. The independent replication of this experiment (second step) confirmed the biological replicability of differential expression (DE) in most of the selected transcripts with notable exceptions of *Hsp23* and *Hsp70*, in which no upregulations were detected in the replicated experiment upon transfer from LD to SD conditions. No or weak gene expression responses to photoperiod or to transfer were seen in NPD-mutants (third step). In NPD strain, the SD1 vs. LD1 analysis was conducted for all 25 transcripts while the T vs. LD analysis was conducted for just 6 selected transcripts that showed strongest responses in the wild-type strain. These results strengthened the view that the differences in gene expression observed in wild-type larvae were associated with photoperiodic programming of their development (SD vs. LD) or with SD-induced developmental switch (T vs. LD). The meaning of individual validation results will be commented below at relevant places. The linear regression analysis (Fig. 3b) shows good correlation between RNAseq results and direct qRT-PCR validation (first step).

## Discussion

In this paper, we first report on profound and characteristic differences in gene expression levels associated with photoperiodic conditions (SD vs. LD), which trigger different developmental destinies (diapause vs. direct development, respectively) in photoperiod-sensitive early 3rd instar wild-type larvae of *C. costata*; secondly, we report on fast, massive and directed change in the gene expression pattern after the transfer of wild-type larvae from LD to SD conditions (developmental switch, T vs. LD). Generally, our results prove that a characteristic transcriptomic signature of diapause develops relatively early in the larval ontogeny, *i.e.* long before overt phenotypic expression of the diapause syndrome. We found at least 1313 differentially expressed (DE) sequences in SD vs. LD wild-type 3rd instars (517 up, 796 down; Fig. 1b). It is problematic to compare the numbers of DE-sequences in various studies differing in experimental design and methodology. Nevertheless, in comparable studies that focused on early stages of diapause, the numbers of SD vs. LD DE-sequences ranged from only 26 (4 up, 22 down) in photoperiod-sensitive pea aphids [50], or 672 (288 up, 384 down) in early embryos of cricket [47], or 704 (336 up, 368 down) in pupae of flesh fly [14], to 2251 or 2506 in the adult females or pharate larvae, respectively, of the Asian tiger mosquito [46, 51]. With the exception of aphids and adult mosquitoes (both sharing rather specific case of maternally induced diapause that is overtly expressed in next generations), however, the above mentioned studies used insects that already *were* in diapause, though in its early stages. In contrast, our study focused on events that are linked to a phase of diapause *induction* that precedes the early phases of diapause development (initiation and maintenance) [6]. It was surprising to see how fast the transcriptomic profiles alter in response to a change of photoperiodic conditions from LD to SD. At T13, which means just one hour after the daylength shortening (advanced light OFF), we registered 903 DE-sequences (421 up, 482 down) in T vs LD conditions. At T17, four hours after the daylength shortening, we found 280 DE-sequences (130 up, 150 down).

Next, we will discuss in more detail the expression responses structured according to biologically meaningful gene clusters representing GO and KEGG categories obtained from our enrichment analysis (Tables 1, 2). We selected the representative sequences for each cluster, those which showed a significant fold-change in relation either to developmental destiny (SD vs. LD) or to developmental switch (T vs. LD) or to both, and present them in a form of heat maps for higher clearness.

### Development: cell cycle

By definition, insect diapause is a centrally regulated arrest, or significant slowdown, of development [1, 6]. In larvae of *C. costata*, the arrest of development is most obviously expressed as a significant slowdown/cessation of the cell proliferation in imaginal discs [7]. The imaginal discs are the pockets of undifferentiated cells that undergo rapid proliferative cell cycles in larvae and later differentiate into adult structures during metamorphosis [55–57]. The size of imaginal discs is very similar in freshly moulted LD and SD 3rd instars [54] as is the relative proportion of CNS cells in the synthetic phase (S phase) of the cell division cycle [7]. The proportion of S cells in CNS gradually grows during the 3rd instar development under LD conditions, while it steadily declines and finally reaches zero under SD conditions (diapause). The CNS cells of diapausing SD larvae are arrested mainly in G0/G1 and partially also in the G2/M phase [7].

**Fig. 4:**
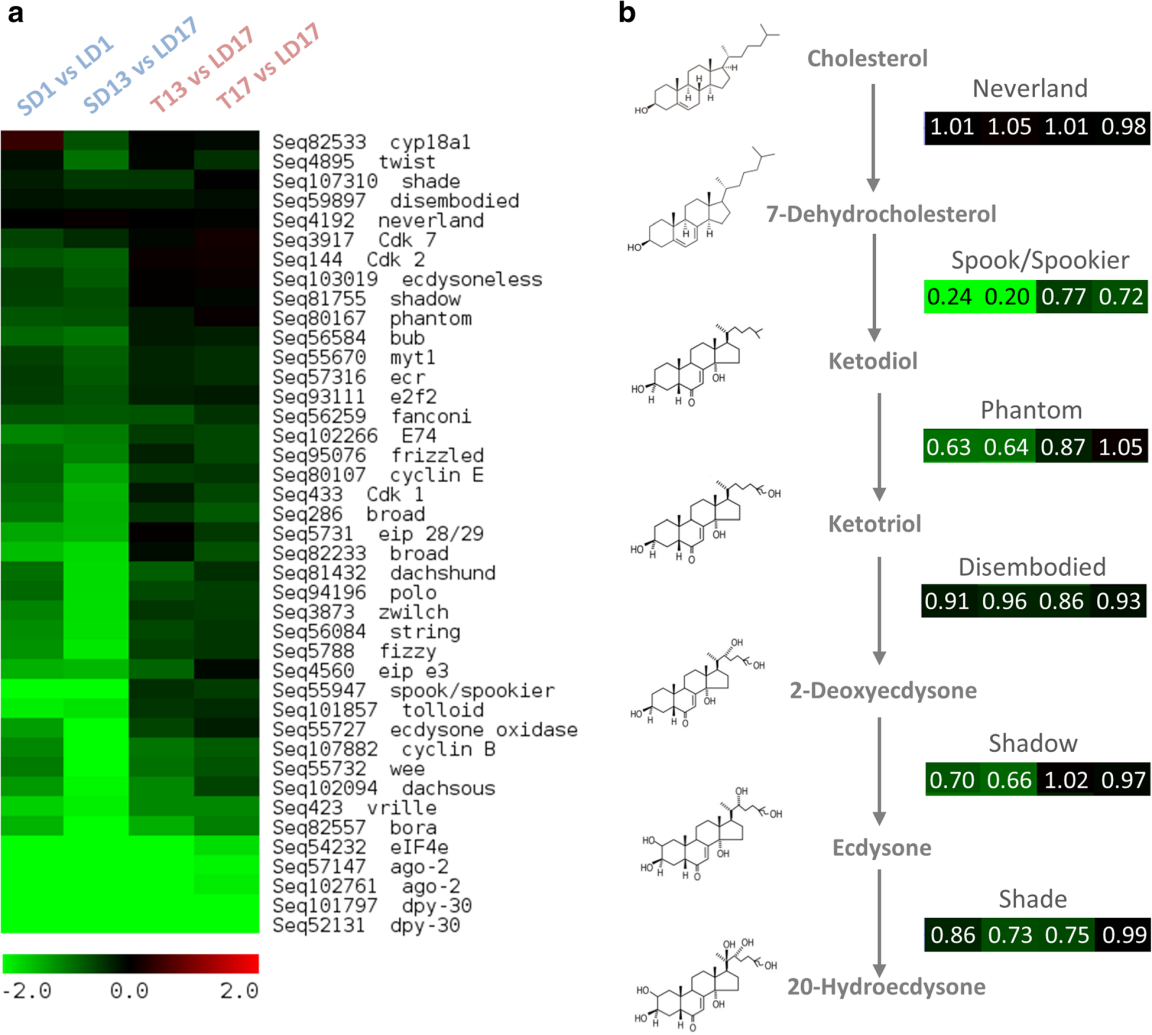
Differential expression of the genes coding for development regulatory factors in larvae of *Chymomyza costata* exposed to Long Day (LD), Short Day (SD) and Transfer (T) from LD to SD photoperiodic conditions. The heat map (**a**) shows log2 fold transformed differences in mRNA relative abundances obtained by DeSEQ2 analysis of RNAseq (Illumina) sequencing data. The schematic depiction (**b**) of biosynthetic pathway for 20-hydroxyecdysone production exhibits differential expression of six selected genes coding for important enzymes shown in a form of small heat maps (organized similarly as in **a**). The numbers in heat maps show fold changes (without log2 transformation)

A developmental arrest under SD conditions was well reflected in our enrichment analysis (Table 1), and it is also visible on the heat map constructed for a cluster of selected genes linked to development, morphogenesis and cell division cycle (Fig. 4a). Most sequences of this cluster were significantly downregulated in wild-type SD larvae compared to LD larvae. Importantly, the overall downregulation trend was clearly confirmed in wild-type larvae in independent validation experiment, while no downregulation was observed in NPD-mutant larvae (Fig. 3), which lack the capacity to respond to SD signal by entering larval diapause [58, 59]. These results indicate that observed differences in gene expression are associated with photoperiodism (present in wild-type, while absent in NPD-mutants). Even more impressive than the difference between SD vs. LD larvae was the fast and directed response of wild-type larvae after transfer from LD to SD conditions (T conditions). Many genes/sequences of the “development” cluster responded by significant downregulation within just one or four hours after transfer (Fig. 4a). Again, we validated the association of such response to photoperiodism (Fig. 3). These results strengthened our earlier observations of very fast responses to T: slowing down the growth of imaginal discs [54] and decreasing the proportion of S phase-cells while increasing the proportion of G2/M cells in larval CNS [7]. In our previous study, the mRNA levels of *dacapo* increased 1.5-fold within one day after T [7]. No response of *dacapo* mRNA to T was observed in the present study (see Sequence ID Seq57069 in Additional file 1: Table S1). *Dacapo*, a homolog of mammalian *p27*, is a potent inhibitor of cell division cycle [60, 61], which makes it a good candidate for mediation of the inhibition of cell cycle in response to shortening of daylength. Our two studies are not, however, directly comparable as they differed mainly in sampled material (CNS or wing discs vs. whole larvae), time resolution (days in earlier study vs. hours in present study), and also in the method of normalization of gene expression data.

Moreover, our results show a number of cell cycle activators among the downregulated sequences. For instance, *cyclin*s *E*, *B* and *Cdk*s *1*, *2*, *7* were downregulated (Fig. 4a). These factors are critical elements of a highly redundant control system which responds to mitogenic signalling and drives the active progression through the cell cycle at two critical checkpoints: Start (G1-S transition) and entry into mitosis (G2-M transition) [62]. The complexations of Cyclin E with Cdk2 and Cyclin A with Cdks 1 and 2 are critical for G1-S transition and S phase progression [63]. The complexation of Cyclin B with Cdk1 is needed for G2-M transition [64] and the Cyclin H with Cdk7 together form activating kinase [65], which again promotes cell cycle progression. Other sequences coding for cell cycle regulators such as *myt1*, *string*, *wee*, *fizzy*, *polo*, *dachshund*, *frizzled*, *dachsous*, *tolloid*, *fanconi*, *bub* and *bora* showed a downregulation response to SD photoperiod and most of them were also downregulated in response to T conditions (Fig. 4a).

Overall, our results confirm that the cell cycle arrest has a central position in diapause syndrome [7, 66–68]. Similarly, positive cell cycle regulators and DNA replication transcripts were downregulated in the adult females of *A. albopictus* during maternal diapause induction phase [51]. In our study, a decrease of expression levels occurred very early in response to T in numerous cell cycle genes. This fast (within four hours of the first extended night) and directed (similar in SD and T conditions) response probably represents the first regulated steps to overt switching from direct development to diapause. These steps are most probably preceded/driven by changes in mitogenic signalling of which ecdysteroid hormones might be an important part [69, 70].

### Development: ecdysteroids

In all arthropods, the ecdysteroids mediate transitions between developmental stages [71]. The ecdysteroids are also very central in regulating many forms of insect diapause [1, 18, 72]. The prohormone ecdysone is synthesized from dietary cholesterol or phytosterols. In larval stages of insects, the biosynthetic pathway is localized in the prothoracic gland (part of a ring gland in larval drosophilids). Ecdysone is released by ring gland and further converted into the active hormone 20-hydroxyecdysone (20-HE) in target tissues [71, 73]. Three distinct peaks (puffs) of ecdysteroid titer can be recognized during the 3rd larval instar of *D. melanogaster* [74]. The last peak, the largest one, occurs in fully grown wandering larvae and it initiates their pupariation [75]. A relatively small middle peak occurs just before the larvae leave the food and exhibit the wandering behaviour [76]. Since neither pupariation nor wandering behaviour occur in diapausing larvae of *C. costata*, we assume that the ecdysteroid peaks stimulating these two transitions are also not expressed in diapause-destined larvae. A first small rise of ecdysteroid levels occurs in *D. melanogaster* already 12 h after ecdysis to the 3rd instar [74–76]. The physiological role of this first peak is poorly characterized but it might be linked to mitogenic signalling to imaginal discs, which first attain competence to metamorphose soon after ecdysis to the 3rd instar [77, 78]. We hypothesize, that this first small ecdysteroid peak, or more precisely the absence of it, might play an important role in inducing larval diapause in *C. costata* as the maximum sensitivity to photoperiodic signal was observed just during the first three days of the 3rd larval instar [54].

We speculate that LD-signal might stimulate synthesis and release of ecdysteroids from early 3rd instar *C. costata*’s ring gland while SD-signal might inhibit it. This hypothesis is indirectly supported by our transcriptomic data, which show that some of the genes coding for enzymes involved in biosynthesis of ecdysone were significantly downregulated in SD vs. LD wild-type 3rd instars (Figs. 3, 4). Most of 20-HE biosynthetic enzymes belong to the family of cytochromes P450 [79, 80]. Some enzymes are well characterized: Neverland, Phantom (CYP306a1), Disembodied (CYP302a1), Shadow (CYP315a1), and Shade (CYP314A1) [81–83]*.* Others, such as two paralogs Spook (CYP370a1) and Spookier (CYP370a2), were only recently identified and their characterization is still incomplete (they are part of the so called ‘black box’) [84, 85]. Only *spookier* gene is expressed in larval ring gland of *D. melanogaster* but two paralogs are probably functionally redundant and they are believed to play a role of rate limiting enzymes for the whole ecdysteroid biosynthetic pathway [85, 86]. Interestingly, the *spook*/*spookier* sequence was considerably (5-fold) downregulated under SD conditions in early 3rd wild-type instars of *C. costata* (Fig. 4b), which was confirmed in an independent experiment but not seen in NPD-mutants (Fig. 4). Moreover, *spook*/*spookier* showed a rapid and statistically significant downregulation (1.3 to 1.4-fold) in response to T (Fig. 4b).

In the target tissues, ecdysteroids bind to ecdysone receptor (EcR) and its dimerization partner ultraspiracle (USP) [1]. The expression patterns of *ecr* and *usp* genes were followed in several diapausing insects and in some but not all of them, a downregulation during diapause was observed: *usp* in pupal diapause of *Sarcophaga crassipalpis* [87]; *ecr* in pupal diapause of *Manduca sexta* [88]. In *C. costata*, *ecr* was dowregulated in SD vs. LD wild-type 3rd instars and also in response to T (Figs. 3, 4) while *usp* showed no significant changes (see Sequence ID Seq2006 in Additional file 1: Table S1). The 20-HE/EcR/USP complex binds DNA and activates the transcription of target genes. In a hierarchical model of the gene transcription regulation [89], the primary-response genes, so called ‘early genes’, encode transcription factors that induce a large set of secondary-response late genes. We found that two of the most important early genes, *broad* and *E74* [90] were significantly downregulated in SD vs. LD and also in T vs. LD wild-type 3rd instars of *C. costata* but not in NPD-mutants (Figs. 3, 4a). Both *broad* and *E74* are induced directly by ecdysone pulses in various larval tissues [66] and serve as key regulators of downstream secondary-response gene expression [91, 92]. Downregulation of early genes during diapause induction in *C. costata* was followed by a reduced expression of some late genes such as *eip 28/29*, *eip e3* (Figs. 3, 4a) or the genes encoding larval serum proteins (*Lsp1* and *Lsp2*) (Fig. 3, Additional file 1: Table S1), which are also under partial control of ecdysteriod puffs [93–98].

Together, our results suggest that inhibition of the 20-HE biosynthetic pathway (downregulation of *spook*/*spookier* expression), and/or downregulation of *ecdysone receptor* (*ecr*), might represent important early steps in diapause induction in *C. costata* larvae, which are translated into the decreased transcription of early and late ecdysone response genes and subsequent blockade of morphogenesis.

### Development: global regulators of gene transcription

One of the most striking responses we have observed in this study was a dramatic and fast downregulation of *dpy-30* under SD conditions and in response to T in wild-type 3rd instar larvae of *C. costata* (Figs. 3, 4a). The *dpy-30* gene encodes an integral subunit of enzymatic complex that is important for global H3-K4 histone methylation in yeast, worms, insects and mammals [95–98]. The histone H3-K4 methylation is generally associated with transcriptional activation [99]. Recently, *dpy-30* was identified as key regulator of the proliferation potential in human primary cells: the DPY-30 depleted cells showed a dramatic proliferation arrest [100] and more than 2-fold downregulation of numerous genes involved in processes such as cellular growth and proliferation, cell cycle, cellular assembly and organisation, DNA recombination, replication and repair [100], which strikingly resembles the processes that we also see downregulated in *C. costata* larvae under SD and T conditions. The DPY-30 directly controls key regulators of cell cycle progression such as ID proteins and E2F transcription factors [100]. Interestingly, *e2f2* sequence was also downregulated in SD vs. LD and T vs. LD wild-type 3rd instar larvae of *C. costata* (Figs. 3, 4a).

Three other factors involved in global regulation of gene expression were found to be strongly downregulated in relation to diapause induction and photoperiodic switch in the wild-type larvae of *C. costata*: the translation initiation factor *eIF4e*; transcription factor *vrille*; and a regulator of response to miRNA *argonaute-2* (*ago-2*) (Figs. 3, 4a). The eIF4E binds the cap structure of the mRNA and initiates its recruitment to the small subunit of ribosome for protein synthesis [101]. Vrille is a bZIP transcriptional factor which influences embryonic development in *D. melanogaster* [102] but is also expressed in imaginal discs and is required for normal cell growth and proliferation [103]. Moreover, Vrille is known to play a role as negative transcriptional regulator in the clock cells of the central circadian pacemaker of *D. melanogaster* [104]. Ago-2 together with Dicer are two pivotal components required for the processing and functioning of small non-coding RNAs (sRNA). While Dicer mediates the cleavage of micro RNA precursors or double-stranded RNA to small 21–23 nucleotide duplexes of siRNA, Ago-2 participates in the formation of RNA-induced silencing complex (RISC) and siRNA binding [105–107]. In addition to its role in RISC complex, Ago-2 influences the patterns of mRNA splicing and binds to specific chromatin sites near gene promoters and negatively regulates the transcription of the Ago-2-associated target genes [108]. The role of sRNA in diapause regulation was recently assayed by [51]. They followed relative abundance of eight genes related to sRNA function in early stages of diapause development in the flesh fly, *Sarcophaga bullata*. Elevated levels of *ago-2* mRNA, and several other genes, were detected in photoperiod-sensitive first instar larvae reared under diapause inducing conditions. Although functional relationships were not directly tested, the differences in transcription patterns between diapause and non-diapause individuals indicated a vital role for sRNA pathways in diapause regulation in *S. bullata* [52].

Collectively, our data suggest that the diapause phenotype in *C. costata* larvae might be importantly regulated by epigenetic processes such as alternative histone marking by methylation (*dpy-30*), alternative splicing and small RNA-mediated regulation of gene expression (*ago-2*), and by factors which influence gene transcription (*e2f2*, *vrille*) and protein translation (*eIF4e*).

### Metabolism: lipids

Insect diapause is typically associated with intensive feeding and building of energy reserves during its early phases (preparation and/or initiation), which is followed by cessation of feeding and metabolic suppression during its later phase (maintenance) [6]. In diapause, the metabolism is rerouted from pathways supporting growth and development to pathways supporting accumulation of reserves and, later, their slow utilization in order to secure basal cellular processes and/or to bolster the processes involved in environmental stress tolerance and long-term survival [9, 10]. These dramatic shifts in feeding behaviour, digestion, and energy utilization are inevitably reflected in a deep alteration of intermediary metabolic pathways [2, 14]. Our enrichment analysis confirmed that various metabolic pathways involving lipids, amino acids and organic acids were significantly upregulated in SD vs. LD larvae of *C. costata* (Table 1). Triacylglycerol depot fats serve as major substrates to fuel metabolism during the period of dormancy in mammalian hibernators as well as in many diapausing insects [109–111]. For instance, the diapause-destined females of the mosquito, *Culex pipiens* switch from blood feeding to seeking sources of nectar to generate lipid reserves required for winter survival [35]. During the preparation for diapause, mosquito females downregulated the genes encoding digestive proteases, while upregulating the *fatty acid synthase* (*fas-1*), a gene associated with accumulation of lipid reserves [35]. In the transition from early to late diapause, however, *fas-1* was only sporadically expressed while numerous other genes involved in fat catabolism were upregulated [37]. Our transcriptomic analysis in *C. costata* did not show any differences in expression of *fas*-like sequence (see Sequence ID Seq95209 in Additional file 1: Table S1). Nevertheless, we observed strong upregulation in SD vs. LD wild-type 3rd instars of several sequences coding for different triacylglycerol (TG) lipases, elongases, desaturases and also for lipophorin receptor. Some of the sequences coding for similar enzymes were rapidly upregulated in response to T (for instance, *lipase 3* CG3635) but the others did not show any response (for instance, *lipase 3* CG8823) or a weak downregulation in response to T (for instance, *lipase 3* CG5932) (Additional file 3: Figure S1A). We cannot provide any integrated functional explanation for individual DE-genes associated with lipid metabolism at this stage of research. The DE just indicates that a metabolic switch to prominent lipid accumulation starts very early during diapause induction. The larvae of both developmental destinies, direct development (LD) and diapause (SD), accumulate lipid reserves during their 3rd instar growth. Nevertheless, SD larvae will finally accumulate more lipids (430 ± 18 μg/mg DM) than their LD counterparts (334 ± 19 μg/mg DM) [109, 112].

### Metabolism: detoxification

A cluster of genes related to detoxification processes was found significantly upregulated in SD vs. LD wild-type 3rd instars in our enrichment analysis (Table 1). Most sequences of this cluster were also upregulated in response to T (Table 2, Additional file 3: Figure S1B). The cluster contains various *cyp* sequences coding for cytochrome P450 enzymes which are known to catalyse not only the biosynthetic reactions of ecdysteroids (discussed above) but most of them are involved in a diverse range of chemical reactions that are important for the detoxification of foreign compounds [113, 114]. It is known that most *cyp* genes are expressed in the larval midgut, Malpighian tubules, and fat body, which is consistent with their protective role against harmful toxic compounds [115]. In addition, the cluster of SD-upregulated sequences contains *UDP-glucuronyltransferase* (*UDP-GT*)*,* various isoforms of *glutathione S-transferase* (*GST*), and *disulfide isomerase*, enzymes known to participate in the removal of foreign metabolites, cell redox homeostasis, and protection against oxidative stress [116].

Some detoxification and antioxidant transcripts such as *ferroredoxin, GST D1*, *cyp12a*, and *cyp6g1* were previously found upregulated in diapausing pupae of *Sarcophaga crassipalpis* [14]. Diapausing insects are typically exposed to various environmental insults including extreme temperatures, desiccation, hypoxia, and immune challenges, which led some authors to expect/explain the activation of cellular protection systems [12]. Our study focuses on very early stages of diapause induction during which the larvae destined for either direct development or diapause share exactly the same environmental conditions (except different photoperiodic regimes). Thus, direct stimulation of protection systems by unfavourable environmental factors can be excluded and our results suggest anticipatory bolstering of the detoxification and antioxidant systems. Additional studies will be needed, however, to determine the functional meaning of the upregulated expression of detoxification genes during diapause induction and to find out whether it also persists until later phases of diapause development in *C. costata*.

### Protein processing in ER: chaperones and heat shock proteins

We found several genes involved in recognition of nascent proteins in endoplasmic reticulum (ER), their quality control, folding end export of misfolded proteins from ER to cytoplasm for proteasome degradation showing an upregulation response specifically to T (Table 2, Additional file 1: Figure S1C). After being produced in the ribosomes, the newly synthesized proteins are transported to the interior of ER via the translocon complex [117] of which a channel protein Sec61 is a main component [118]. The TRAP and TRAM proteins are further known components of the translocon complex [119]. The lectin CRT (Calreticulin) is one of the chaperones that bind the newly synthesized protein in ER and assist in its folding and quality control [120]. Other ER luminal chaperones and co-chaperones, such as NEF, BiP (Hsc70-3), GrP94 (Hsp83), and Dnaj (Hsp40) are hierarchically organized in processes of protein recognition and folding [121, 122]. The family of protein disulfide isomerases (PDIs) catalyzes the formation and isomerization of disulfide bonds [122]. Misfolded proteins must be recognized and removed, which is achieved by their targeting for ER associated protein degradation (ERAD) and subsequent retrotranslocation to cytosol [123]. Proteins recognized by sensors (such as BiP and PDIs) as misfolded are transported into the cytosol via poorly characterized system, which probably includes again the Sec61 channel protein in addition to other members [123, 124]. In the cytosol, another suite of chaperones, such as Hsp70, Hsp83, Hsp40, and Hsp23 recognize hydrophobic patches of misfolded proteins and may either refold them or direct them for further proteasome degradation [125].

The sequences coding for all the proteins that were mentioned in the above paragraph were found upregulated in wild-type *C. costata* larvae in response to T conditions (transfer from LD to SD) (Additional file 3: Figure S1C). In contrast, slight downregulations of *Hsp70* and *Hsp23* mRNA levels in response to T were observed in NPD mutants (Fig. 4). Though our replicated experiment failed to confirm the upregulations of *Hsp70* and *Hsp23* mRNA levels in the wild-type strain (Fig. 4), the upregulations of *sec61* and *CRT* sequences representing early steps in ER processing were confirmed (Fig. 4). These results might indicate a critical influence of sample timing. A typical Hsps transcriptional response to stress is known to have very rapid dynamism [126]. However, our variable results suggest that additional experiments are needed to clarify to what extent the upregulation of gene cluster of ER processing and heat shock response was linked to photoperiodic diapause induction and to differentiate this from mere artefacts caused by inevitable manipulation stress.

The upregulation of heat shock proteins (Hsps) during insect diapause emerges as a common pattern across species [127–129]. The elevated levels of Hsps observed during diapause were most often explained in terms of increased cold tolerance, developmental suppression, cytoskeleton stabilization or protein sequestering (see [127] for discussion). In our study, we did not see any differences between direct development-destined (LD) and diapause-destined (SD) larvae but we rather observed a very rapid response to T (developmental switch), at least in the RNAseq experiment and its direct validation by qPCR. Hypothetically, such activation of a whole pathway for processing of the newly synthesized proteins in ER (if confirmed by follow-up experiments) might serve for rapid removal of protein species which are no more compatible with newly initiated developmental destiny after switching from direct development to diapause (T conditions).

### Cuticle

Diapause-destined wild-type larvae of *C. costata* were enriched in sequences coding for genes related to development of larval cuticle (Table 1) and the same cluster appeared upregulated in response to T (Table 2, Additional file 3: Figure S1D). These responses were confirmed in an independent experiment and were not observed in NPD-mutants (Fig. 3). Cuticular proteins (CPs) form a very diverse ‘superfamily’ [130] of mainly structural components of arthropod integument, which are synthesized in epidermal cells according to a temporal pattern of moults [131]. CPs are physically interconnected with chitin and stabilized (sclerotized) by crosslinking quinones [132] to form the layered structure of cuticle, which protects the soft insect body against all biotic and abiotic external factors. The diapause individuals are designed for much longer survival than their non-diapause counterparts and will be exposed to different environmental stressors. Therefore, the cuticle of diapause insect probably requires compositional modifications, which are reflected in differential regulation of specific CPs and related pathways. As the massive synthesis of CPs is linked to moulting, the expression of CPs genes might be regulated by changes in titres of developmental hormones [133, 134]. Differential regulation of CPs and the cuticle sclerotization pathway by changes in photoperiod was previously described in aphids [50, 135, 136] or in the mosquito, *Culex pipiens* [137]. It was also previously shown that the total amount and composition of another cuticular component (cuticular hydrocarbons) differ between non-diapause and diapause insects [138–141].

## Conclusions

Our RNAseq study reveals strong candidate genes (bracketed in following text) that might be principal regulators of very early stages of photoperiodically induced diapause in larvae of *C. costata* and, possibly, in other insects entering diapause in late larval instars. Our results indicate that the short-day signal inhibits the enzymatic pathway (*spook*/*spookier*) for the synthesis of an important developmental hormone, 20-hydroxy ecdysone, which eliminates a small peak of ecdysone titer typically occurring during early 3rd instar ontogeny under long days. In addition, expression of ecdysone receptor (*ecr*) is downregulated under short days. These changes are probably translated into decreased transcription of early (*broad* and *E74*) and late (for instance, *eip 28/29*, *eip e3*) ecdysone response genes, which, in turn, may govern subsequent deep alteration of gene expression profile and lead to diapause phenotype. Some factors with broad influence on gene transcription (*e2f2*, *vrille*) and protein translation (*eIF4e*) are strongly downregulated under short days. Epigenetic processes such as alternative histone marking by methylation (*dpy-30*), alternative splicing and small RNA-mediated regulation of gene expression (*ago-2*), and perhaps also ER processing and ERAD degradation of newly synthesized proteins, emerge as potentially important elements of the developmental switch. Blockade of morphogenesis (principal feature of diapause) is clearly represented in our results as a general decrease of expression levels in numerous mRNAs coding for cell cycle regulators. Restructuring of metabolic pathways, another feature of diapause, is indicated by differential expression of genes involved in metabolism of lipids, amino acids, organic acids, detoxification, redox balance, protection against oxidative stress, cuticle formation and synthesis of larval storage proteins.

One of the most salient outcomes of our study is that the highly complex alteration of gene transcription was observed within a time frame of only four hours after the change of photoperiodic signal (in fact, just in response to advanced dusk time without exposing the insects to even single complete long night). The response was not only highly complex and fast but also clearly directed (in contrast to undirected disturbance), which was documented by similarity of gene expression responses to constant photoperiod (short days during whole larval development) and change of it (transfer to short days). Moreover, we were able to verify the relevance of our RNAseq differential gene expression results in an independent qRT-PCR experiment involving wild-type (photoperiodic) and NPD-mutant (non-photoperiodic) strains of *C. costata*. The transfer from LD to SD conditions is known to gradually but rapidly (within 3–5 long nights) switch the ontogenetic programming from direct development to diapause [7, 52, 53]. In this paper, we show that the regulated change of developmental pathway starts as early as 1 h after the shortening of daytime. Such rapid change from direct development to diapause may reflect high ecological importance of diapause for *C. costata* larvae: missing the diapause entry at ecologically appropriate time (under short days signalling for coming winter) would mean strongly decreased chance for winter survival (risking the *loss of life*). Therefore, even the larvae that are very close to pupariation are able to rapidly switch for diapause if the photoperiod “seems to signal for it” (responding already to a phase shift of dusk time or to the first long night). The “wrong” decision to enter diapause may be easily corrected anytime later by returning to direct development pathway (risking the *loss of time* only) as the diapause larvae maintain photoperiodic sensitivity for many months (until death) when exposed to permissive ambient temperatures such as 18 °C [53].

In conclusion, our study indicates several strong candidate genes that deserve detailed attention in future functional studies. These candidates might represent upstream general regulators of a complex transfiguration of gene expression pattern, which eventually leads to phenotypic switch of developmental programming from direct ontogeny to larval diapause in insects.

## Methods

### Insect rearing, diapause induction and sampling

**Fig. 5:**
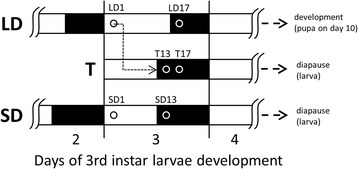
Schematic depiction of experimental design. Larvae of *Chymomyza costata* were sampled on day 3 of their 3rd instar, which corresponds to the stage of maximum sensitivity to photoperiodic signal [54]. Larvae were exposed to different photoperiodic conditions: constant Long Days (LD) since embryonic stage promoting direct development to pupa (pupariation on day 10 in average); constant Short Days (SD) since embryonic stage inducing larval diapause (initiated between days 10 and 40); and Transfer (T) from LD to SD switching the direct development to diapause. The samples were taken at two Zeitgeber times in each photoperiodic conditions (*i.e.* LD1 and LD13; SD1 and SD17; T13 and T17). See text for more explanations

Two strains of *Chymomyza costata* (Zetterstedt, 1838) (Diptera: Drosophilidae) were used in this study: a wild-type (Sapporo) strain originally collected in Sapporo, Hokkaido, Japan, in 1983 and a non-photoperiodic-diapause (NPD) mutant strain, which was isolated by [58] from wild-type flies collected in Tomakomai, Hokkaido, Japan. Insects were cultured at constant temperature of 18 °C in incubators MIR154 (Sanyo Electric, Osaka, Japan) on an artificial diet as described by [142]. Developmental destiny of larvae was programmed using two different photoperiodic regimes: a long day regime (LD, 16 h light: 8 h dark) at which all larvae of both strains continue direct development (*i.e.* pupariate, pupate and metamorphose to adults), and a short day regime (SD, 12 h light: 12 h dark) that induces larval diapause in all individuals of Sapporo strain but no-diapause in larvae of NPD strain [58]. In order to switch the developmental destiny from direct development to diapause, the LD-reared wild-type larvae were transferred to SD conditions (T, transfer) on day three of their 3rd larval instar (*i.e.* approximately when 15-day-old since the egg deposition). The transferred larvae experienced four hour advanced light OFF time (dusk) on day 15 (see scheme in Fig. 5). All wild-type strain larvae respond to such transfer by switching the developmental destiny from direct development to diapause, while NPD-mutant larvae show no response and continue developing [54].

All experimental insects came from synchronized eggs (laid within one day) and a second developmental synchronization (for details, see [54]) took place during the 2nd to 3rd instar transition that means three days prior to sampling. All samples were taken on day three of 3rd instar when the larvae reach their maximum sensitivity to photoperiodic signal [53]. The samples were taken at two Zeitgeber times (Zt’s) in each photoperiodic regime (i.e. LD1 and LD13; SD1 and SD17; T13 and T17, see Fig. 5). The given Zt’s were selected on purpose, first, in order to take into account potential effect of diurnal fluctuations, which we know is generally very small based on the results of our parallel study (Koštál and Schöttner, unpublished results), and second, to allow comparing the transcriptomic responses to switch OFF the light (phase shift of dusk time). Three biological replicates were taken at each Zt, and each replicate consisted of a pool of 10 larvae. Night samples were taken under dim red light. Larvae were collected to 400 μl of ice cold RiboZol RNA Extraction Reagent (Amresco, Solon, OH, USA) in 1.5 ml microvial, immediately cut to small pieces by scissors and stored at −80 °C until next processing.

### RNA sample processing, cDNA library production and RNAseq

The total RNA was extracted from whole larvae using the RiboZol RNA Extraction Reagent. Pellet of total RNA was dissolved in 20 μl of DEPC-treated water and an aliquot of 5 ul was taken for total RNA quality assessment on denaturing agarose gel and concentration measurement using Cary50 UV–VIS spectrophotometer (Varian, Palo Alto, CA, USA). The total RNA concentrations were levelled exactly to 0.5 ug/ul and the samples were either sent to the EMBLGenomics Core Facilities (GeneCore, Heidelberg, Germany) for cDNA library production and Illumina RNAseq or used for qRT-PCR validation of RNAseq results.

The cDNA libraries were prepared from the samples of Sapporo strain larvae using Covaris S2 (Covaris, Woburn, Massachusetts, USA) for fragmentation aiming for an insert size of about 150 nt and TruSeq RNA sample prep kit (Illumina, San Diego, California, USA).

The cDNA libraries were then sequenced using HiSeq2000 sequencer (Illumina, San Diego, California, USA). For the *de-novo* transcriptome assembly, the cDNA library was run on two lanes using 100 nt paired end sequencing, while for transcriptome profiling in the main experiment, we ran the samples randomly distributed on 3 lanes using 50 nt single end sequencing.

### De-novo transcriptome assembly, gene annotation and mapping

For the *de-novo* transcriptome assembly, we used a mix of 18-d-old larvae that were collected at 6 different Zt’s (1, 5, 9, 13, 17, 21) under both, LD and SD conditions in order to make sure that we do not miss any gene related to different developmental destinies or different Zt’s. The quality of RNAseq results was first assessed using FastQC (http://www.bioinformatics.babraham.ac.uk/projects/fastqc/). The raw reads were trimmed and all adapters and overrepresented sequences were removed with Trimmomatic software [143]. The resulting reads were filtered with a Phred quality score of at least 28 and with a read length of at least 100 bp. The transcriptome was then assembled using kmers 35, 55, 75 in SOAPdenovoTrans [144] using default settings and merged with cd-hit-est with a setting of 100 % identity, to get rid of nested sequences [145]. A minimum sequence size was set to 100 nt in order to not miss short sequences. The transcriptome assembly was then annotated using Blastx (RefSeq NCBI database, E-value below 0.001). GO-terms, INTERPROs and Kegg IDs were retrieved using Blast2GO [146]. Since the exact number of genes/transcripts in *C. costata* is unknown, we blasted our database against *D. melanogaster* transcriptome in order to find putative orthologous genes using a minimum identity threshold of 50 % and an E-value below 1e-10. The *D. melanogaster* transcriptome was chosen primarily because of its very good annotation and also because of its close taxonomic relationship to *C. costata*.

### Differential expression analysis, GO-term enrichment analysis, functional clustering and statistical analysis of RNAseq data

Quality control of RNAseq results was conducted as described above. Reads were then mapped to our *C. costata* reference transcriptome developed previously using Bowtie 2 [147] with default parameters. Read counts were estimated with eXpress (http://bio.math.berkeley.edu/eXpress/overview.html) and the differential expression analysis was conducted using DeSEQ2 [148]. Only those transcripts, which showed a log2 fold change (FC) above 0.55 or below −0.55 (equivalent to 1.5 absolute fold change) and a corrected *P*-value (using Benjamini and Hochberg multiple testing correction) below 0.01 were considered as significantly differentially expressed (DE). We only considered the sequences with a baseMean above 10 in order to avoid false positive genes (baseMean reflects the mean number of sequencing reads all across the sample per sequence). Using DESeq2, the normalization of the RAW counts has been conducted by taking into account the library sizes. Several basic comparisons were made: Short day vs. Long day (SD1 vs. LD1 and SD13 vs LD17; *i.e.* differences associated with developmental destiny) and Transfer vs. Long day (T13 vs. LD17 and T17 vs LD17; *i.e.* differences associated with developmental switch). To detect if any particular biological process, molecular function or cellular component were enriched in any condition, GO-term enrichment analyses were conducted using the *Drosophila* orthologous gene IDs with the module ClueGO [149] on Cytoscape software [150] and compared against the whole *Drosophila melanogaster* transcriptome. If several contigs were sharing the same Blast X result, then only one sequence would be considered for the enrichment analysis removing any redundancy in the dataset. The GO terms were considered significantly over- or underrepresented when a corrected Bonferroni *p*-value was below 0.05.

Based on our GO term enrichment analysis, representative and candidate sequences/transcripts were selected and clustered according to their putative role in diapause. Heat maps representing the differential expression of the gene clusters were constructed with TMev using Euclidian metric (average distance) [151].

### Validation of RNAseq results

We validated and verified our results at three levels using qRT-PCR analysis of 25 selected mRNA transcripts (Additional file 4: Table S3). In the first level, the aliquots of total RNA that was subjected previously to the RNAseq experiment were taken for direct validation of the RNAseq results. The 5 uL (2.5 ug) aliquots of total RNA were treated with DNase I (Ambion, Life Technologies) followed by the first strand cDNA synthesis using Superscript III (Invitrogen, Carlsbad, CA, USA). The cDNA products (20 μL) were diluted 25 times with sterile water. Relative abundances of mRNA transcripts for selected transcripts were measured by quantitative real time PCR (qPCR) using the CFX96 PCR light cycler (BioRad, Philadelphia, PA, USA) and the IQ SYBR Green SuperMix (Bio-Rad). PCR reactions (total volume of 20 μL) contained 5 μL of diluted cDNA template and were primed with a pair of gene-specific oligonucleotide primers (Additional file 4: Table S3), each supplied in a final concentration of 400 nM. Cycling parameters were 3 min at 95 °C followed by 40 cycles of 94 °C for 15 s, 60 °C for 30s and 72 °C for 30s. Analysis of melt curves verified that only one product was amplified in each reaction. In addition, we checked the size of the PCR products for each gene by electrophoresis on 2 % agarose gel in selected samples. Emission of a fluorescent signal resulting from SYBR Green binding to double-stranded DNA PCR products was detected with increasing PCR cycle number. Quantitation cycle (*C*_Q_) for each sample was automatically calculated using the algorithm built in the CFX96 PCR light cycler software. The levels of mRNA transcripts of *Ribosomal protein L32* (*Rpl32*) and *β-tubulin 56D* (*β-tub*) served as endogenous reference standards for relative quantification of the target transcript levels [152]. Each sample was run as a doublet (two technical replicates) of which the mean was taken for calculation. Relative ratios of the candidate mRNA levels (*C*_Q_) to geometric mean of the levels (*C*_Q_) of two reference gene mRNAs were calculated according to [153]. The Log2-transformed relative ratios were statistically analyzed using one-way ANOVA (with confidence intervals set to 95 %) followed by Bonferroni post-hoc tests using Prism6 (GraphPad Software, San Diego, CA, USA).

Second level: In order to verify that the differences in gene expression observed in RNAseq experiment are based on robust biological background, we conducted an independent replication of the experiment and sampled new wild-type larvae from different generation (1 year later). The same experimental design (LD, SD and T) was applied as for original RNAseq experiment, except that we omitted sampling at LD Zt1 and SD Zt1, and all samples were processed for qRT-PCR analysis.

Third level: The NPD-mutant strain larvae were used in order to see whether the differences in gene expression observed in RNAseq experiment are specifically related to sensitivity to photoperiodic signal. The NPD-mutant strain larvae (insensitive to photoperiodic signal) were subjected to the same experimental design (LD, SD and T) as wild-type larvae in original RNAseq experiment and all samples were processed for qRT-PCR analysis.

## Additional files

Additional file 1:
**Table S1.** List of 21,327 putative *Chymomyza costata* mRNA transcripts obtained by *de novo* transcriptome assembly of HiSeq2000 (Illumina) sequencing data. (XLSX 5626 kb) Additional file 2:
**Table S2.** List of sequences commonly found differentially expressed in larvae of *Chymomyza costata* during Night vs. Day under both photoperiodic conditions, Long Day and Short Day (LD and SD). (DOCX 17 kb) Additional file 3:
**Figure S1.** Differential gene expression in larvae of *Chymomyza costata* exposed to Long Day (LD), Short Day (SD) and Transfer (T) from LD to SD photoperiodic conditions. The heat maps show log2 fold transformed differences in mRNA relative abundances obtained by DeSEQ2 analysis of RNAseq (Illumina) sequencing data. Genes representing four functional clusters identified by enrichment analysis: (A) lipid metabolism; (B) detoxification metabolism; (C) protein processing in ER, chaperones and heat shock proteins; and (D) cuticle development. (DOCX 175 kb) Additional file 4:
**Table S3.** List of sequences and gene-specific primers selected for three-step validation process of RNAseq differential gene expression results. (DOCX 19 kb)

## Abbreviations

DE: Differentially expressed
20-HE: 20-hydroxy ecdysone
LD: Long days
NPD: Non-photoperiodic strain of *C. costata*
SD: Short days
T: Transfer from LD to SD
Zt: Zeitgeber time
