# Supplementary material for: Early transcriptional events linked to induction of diapause revealed by RNAseq in larvae of drosophilid fly, Chymomyza costata
Source: BMC Genomics. 2015 Sep 21;16:720. doi: 10.1186/s12864-015-1907-4 (PMC4578651; doi:10.1186/s12864-015-1907-4)
Supplement: Additional file 2: — Table S2. List of sequences commonly found differentially expressed in larvae of Chymomyza costata during Night vs. Day under both photoperiodic conditions, Long Day and Short Day (LD and SD). (DOCX 17 kb) [file 12864_2015_1907_MOESM2_ESM.docx]

**Additional file 2: Table S2.** List of sequences commonly found differentially expressed in larvae of *Chymomyza costata* during Night vs. Day under both photoperiodic conditions, Long Day and Short Day (LD and SD).

|  |  |  |  | Night vs Day (LD) | |  | Night vs Day (SD) | |
| --- | --- | --- | --- | --- | --- | --- | --- | --- |
| Sequence ID | Sequence full description (according to BLAST results) | *D. melanogaster* orthologous transcript |  | Fold change | *P* adj * |  | Fold change | *P* adj * |
|  |  |  |  |  |  |  |  |  |
| Seq78517 | CG34227 | CG34227-RA |  | 3.19 | 4.41877E-05 |  | 3.05 | 0.000923732 |
| Seq423 | Vrille | CG14029-RD |  | 2.07 | 5.55622E-05 |  | 1.72 | 0.033561614 |
| Seq60022 | sosie | CG13636-RA |  | 1.98 | 6.69996E-05 |  | 1.80 | 0.044266382 |
| Seq5158 | sosie | CG13636-RA |  | 1.85 | 0.000302545 |  | 2.02 | 0.012356571 |
| Seq83275 | polya-binding protein interacting protein isoform b | CG12358-RA |  | 1.55 | 0.004047332 |  | 1.51 | 0.017058928 |
|  |  |  |  |  |  |  |  |  |
| Seq93228 | esterase b1-like | CG1082-RA |  | 0.66 | 0.020384284 |  | 0.65 | 0.025065369 |
| Seq53939 | cuticle | CG2342-RA |  | 0.63 | 0.049195753 |  | 0.58 | 0.017058928 |
| Seq102268 | acyl- delta desaturase-like | CG8630-RA |  | 0.62 | 0.023079796 |  | 0.62 | 0.029173921 |
| Seq107229 | ---NA--- | NA |  | 0.61 | 0.020769233 |  | 0.62 | 0.041795212 |
| Seq91954 | pupal cuticle protein g1a-like | CG13063-RA |  | 0.58 | 0.025841368 |  | 0.38 | 9.59463E-06 |
| Seq79202 | troponin isoform 3-like | CG9073-RA |  | 0.56 | 0.001070449 |  | 0.61 | 0.017058928 |
| Seq79638 | fibrous sheath cabyr-binding | CG11131-RB |  | 0.56 | 0.002046314 |  | 0.62 | 0.030415355 |
| Seq113304 | larval cuticle protein 8-like | CG6956-RA |  | 0.56 | 0.000884282 |  | 0.61 | 0.017664823 |
| Seq53394 | a-kinase anchor protein 14-like | NA |  | 0.54 | 3.75626E-06 |  | 0.65 | 0.010604606 |
| Seq51747 | larval cuticle protein 5-like | CG15515-RB |  | 0.54 | 0.000620732 |  | 0.60 | 0.017058928 |
| Seq92034 | CG13063 | CG13063-RA |  | 0.54 | 0.000193722 |  | 0.65 | 0.030415355 |
| Seq24459 | ---NA--- | NA |  | 0.53 | 0.003806955 |  | 0.49 | 0.003043169 |
| Seq54609 | fibrous sheath cabyr-binding | CG13033-RA |  | 0.46 | 0.000776035 |  | 0.56 | 0.01276934 |
| Seq4959 | pupal cuticle protein edg-78e-like | CG18349-RA |  | 0.41 | 1.9114E-07 |  | 0.58 | 0.014504997 |
| Seq105165 | ---NA--- | NA |  | 0.25 | 9.9234E-12 |  | 0.48 | 0.004311135 |
| Seq78737 | larval cuticle protein 8-like | CG10534-RA |  | 0.24 | 1.01009E-11 |  | 0.47 | 0.010875784 |
| Seq52196 | ---NA--- | NA |  | 0.23 | 0.000409758 |  | 0.40 | 0.014504997 |
| Seq1794 | larval cuticle protein 8-like | CG6956-RA |  | 0.20 | 0.000325109 |  | 0.35 | 0.003327867 |

* Fold changes adjusted *P* values (*P* adj) were obtained after conducting the DESeq2 package.
