# Supplementary material for: Early transcriptional events linked to induction of diapause revealed by RNAseq in larvae of drosophilid fly, Chymomyza costata
Source: BMC Genomics. 2015 Sep 21;16:720. doi: 10.1186/s12864-015-1907-4 (PMC4578651; doi:10.1186/s12864-015-1907-4)
Supplement: Additional file 3: — Figure S1. Differential gene expression in larvae of Chymomyza costata exposed to Long Day (LD), Short Day (SD) and Transfer (T) from LD to SD photoperiodic conditions. The heat maps show log2 fold transformed differences in mRNA relative abundances obtained by DeSEQ2 analysis of RNAseq (Illumina) sequencing data. Genes representing four functional clusters identified by enrichment analysis: (A) lipid metabolism; (B) detoxification metabolism; (C) protein processing in ER, chaperones and heat shock proteins; and (D) cuticle development. (DOCX 175 kb) [file 12864_2015_1907_MOESM3_ESM.docx]

**Additional file 3: Figure S1.** Differential gene expression in larvae of *Chymomyza costata* exposed to Long Day (LD), Short Day (SD) and Transfer (T) from LD to SD photoperiodic conditions. The heat maps show log2 fold transformed differences in mRNA relative abundances obtained by DeSEQ2 analysis of RNAseq (Illumina) sequencing data. Genes representing four functional clusters identified by enrichment analysis: (A) lipid metabolism; (B) detoxification metabolism; (C) protein processing in ER, chaperones and heat shock proteins; and (D) cuticle development.

**T17 vs LD17**

**SD1 vs LD1**

**SD13 vs LD17**

**T13 vs LD17**


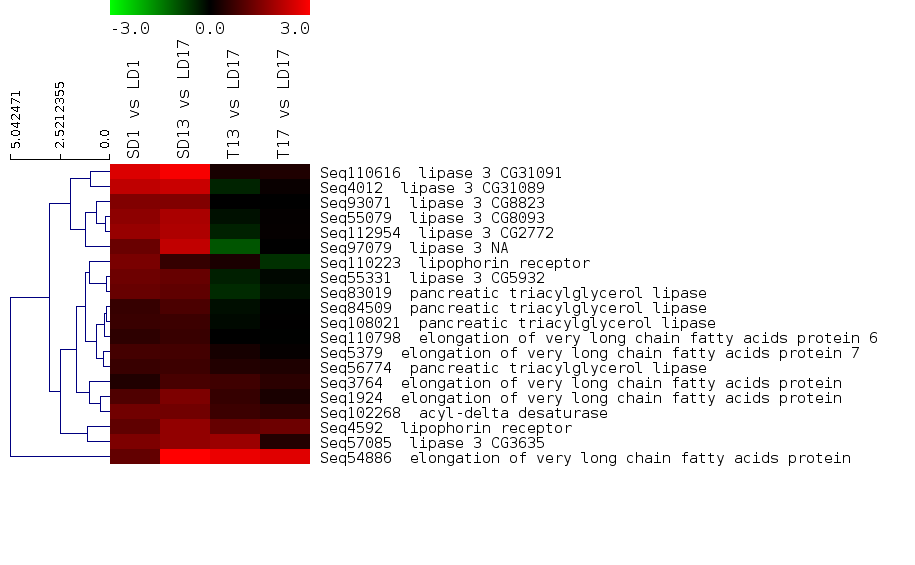


**A**

LIPIDS


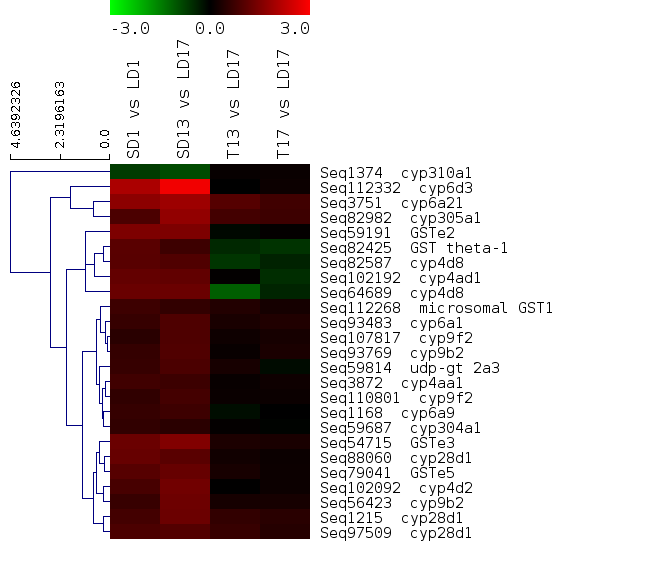


**B**

DETOXIFICATION


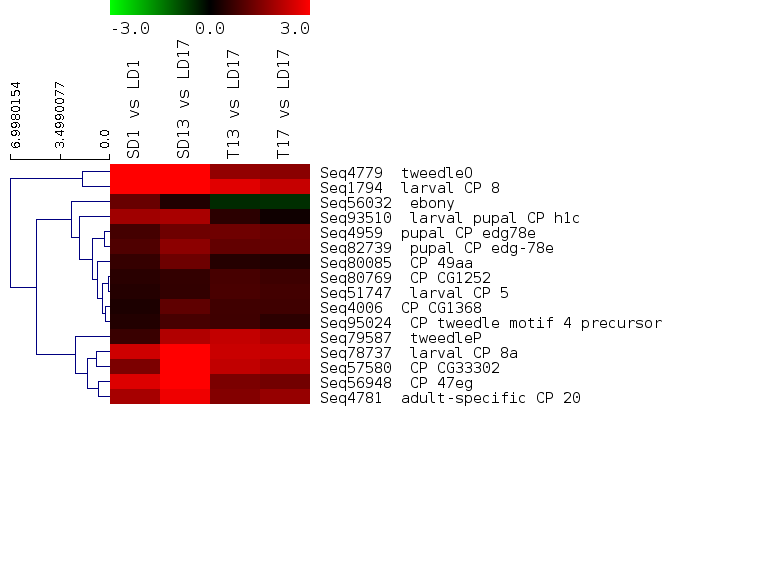

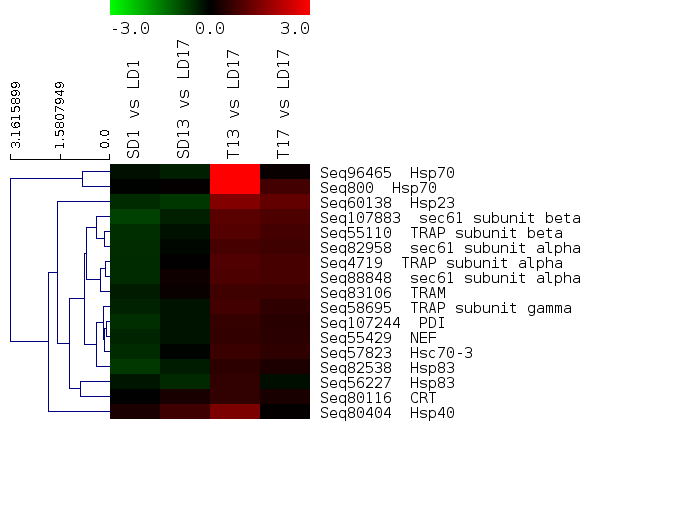


CUTICLE

**D**

ER PROCESSING & HSPs

**C**

**SD13 vs LD17**

**SD1 vs LD1**

**T13 vs LD17**

**T17 vs LD17**
