# Supplementary material for: Early transcriptional events linked to induction of diapause revealed by RNAseq in larvae of drosophilid fly, Chymomyza costata
Source: BMC Genomics. 2015 Sep 21;16:720. doi: 10.1186/s12864-015-1907-4 (PMC4578651; doi:10.1186/s12864-015-1907-4)
Supplement: Additional file 4: — Table S3. List of sequences and gene-specific primers selected for three-step validation process of RNAseq differential gene expression results. (DOCX 19 kb) [file 12864_2015_1907_MOESM4_ESM.docx]

**Additional file 4: Table S3.** List of sequences and gene-specific primers selected for three-step validation process of RNAseq differential gene expression results

| Sequence ID | Gene | Primer sequence (5' -> 3') | Product length (bp) |
| --- | --- | --- | --- |
| Seq78517 | *CG 34227* | CGTGCTTTGTTTGGCCTACG | 93 |
|  |  | AGAAGACTGACAGCAGCGAC |  |
|  |  |  |  |
| Seq78737 | *larval CP 8a* | GCTATCTCCGTCAAGGGCTC | 88 |
|  |  | GGAAACCGTTCTCATCAGCG |  |
|  |  |  |  |
| Seq55814 | *glutactin* | CATTGGGCTCGTATCGGTCT | 190 |
|  |  | CGCGCTTTCTGTTGGTTTCT |  |
|  |  |  |  |
| Seq55334 | *Obp 83* | TACTGATCGAGAGCATGCCG | 145 |
|  |  | CGCGTAAAGCAGGGTATGGG |  |
|  |  |  |  |
| Seq55855 | *Lsp 2* | GATCAAGGCGCGTCAATCTC | 200 |
|  |  | CACCAGCAACCAGATCCACT |  |
|  |  |  |  |
| Seq94976 | *takeout* | AAAAGGCCCCTACTCAATCG | 196 |
|  |  | CTTGTATGAAACGTGTGCAGG |  |
|  |  |  |  |
| Seq55947 | *spook/spookier* | GGGCTCTGATTCTGGTGTAGAG | 180 |
|  |  | GTCCGTTTTCCAATACTGAAGGG |  |
|  |  |  |  |
| Seq60189 | *cyclin E* | CTGCTGGGAGCACTGAACTG | 148 |
|  |  | AGCCCGAAAACTGCGGATAG |  |
|  |  |  |  |
| Seq433 | *Cdk 1* | ACCGGTACGCATTTACACC | 154 |
|  |  | ACCTTGAAAAAGTGGCTTGC |  |
|  |  |  |  |
| Seq54571 | *Will die slowly* | CTCACCTCGAGTCTGAACAGC | 159 |
|  |  | ACTCTTATCCGTACTGCCCG |  |
|  |  |  |  |
| Seq57316 | *ecr* | AGAGCTACTACATCGACACGC | 147 |
|  |  | GCGAGAAGCACATTTCGGC |  |
|  |  |  |  |
| Seq54232 | *eIF4e* | AATAGCAAAGGTGGGCGCTG | 192 |
|  |  | GTTAAAGCCATCGGCAGTCC |  |
|  |  |  |  |
| Seq102266 | *E74* | GCAGTCGCGAAGGATCTACC | 124 |
|  |  | TCGACCAGCTTGAATACGCC |  |
|  |  |  |  |
| Seq4560 | *eip e3* | AGACTTTGATCGCCTACCGC | 197 |
|  |  | GCACACGTTTCTTACCAGCC |  |
|  |  |  |  |
| Seq102486 | *ago-2* | TCCAAAAGGCGAAGGAGACG | 155 |
|  |  | CGTTGTAACGAGTTGGCCTG |  |
|  |  |  |  |
| Seq101405 | *dpy-30* | CATGCACTCGATGAGGTGGG | 88 |
|  |  | TGCAGCGATGCTTGTTCTTTAG |  |
|  |  |  |  |
| Seq107992 | *broad* | TGAGGGTCGCAGCATAAAGG | 143 |
|  |  | CCACCAGCGAGTGTAGATCC |  |
|  |  |  |  |
| Seq423 | *vrille* | TGATGGCGGTGATAACTCCAG | 114 |
|  |  | ACTGCGCTCACTACCTTGG |  |
|  |  |  |  |
| Seq60138 | *Hsp23* | TGAGCGAATTCAAGCCCAATG | 179 |
|  |  | AGAGCTTAAGGTGGAGATGGC |  |
|  |  |  |  |
| Seq93839 | *Hsp70* | TTAAGCGGCGATCAGAGTGG | 94 |
|  |  | TCATAACGCCTCCAGCTGTC |  |
|  |  |  |  |
| Seq55110 | *TRAP* | CACCGTGGAACGTATTGCTC | 173 |
|  |  | TGACAATTCCACCCTCACCAG |  |
|  |  |  |  |
| Seq80116 | *CRT* | TCTGCGTAAGGAAGTTTGCAC | 108 |
|  |  | GCCTTTGAAGCCAACTCGATG |  |
|  |  |  |  |
| Seq82958 | *sec61 subunit alpha* | CAGCTCAGCCAAGGATGTTG | 187 |
|  |  | ACCAGTGCCCGAACCAATAG |  |
|  |  |  |  |
| Seq107883 | *sec61 subunit beta* | TCCCGGCATTAAAGTTGGTC | 84 |
|  |  | CCCCAAATGTGCAGCATAAAC |  |
|  |  |  |  |
| Seq107882 | *cyclin B* | ATCGCGTCCACTGCCAATAC | 197 |
|  |  | CGGCCTTGGCATTACCATTTAG |  |
